# The relationship between school physical activity policy and objectively measured physical activity of elementary school students: a multilevel model analysis

**DOI:** 10.1186/2049-3258-72-20

**Published:** 2014-06-16

**Authors:** Guy Faulkner, Laura Zeglen, Scott Leatherdale, Steve Manske, Michelle Stone

**Affiliations:** 1 Faculty of Kinesiology and Physical Education, University of Toronto, 55 Harbord Street, Toronto, ON M5S 2W6, Canada; 2 School of Public Health and Health Systems, University of Waterloo, 200 University Ave West, Waterloo, ON N2L 3G1, Canada; 3 School of Health and Human Performance, Faculty of Health Professions, Dalhousie University, 6230 South Street, PO Box 15000, Halifax, NS B3H4R2, Canada

## Abstract

**Background:**

There is evidence of school level variability in the physical activity of children and youth. Less is known about factors that may contribute to this variation. The purpose of this study was to examine if the school health environment (Healthy Physical Environment, Instruction and Programs, Supportive Social Environment, and Community Partnerships) is associated with objectively measured time spent in light to vigorous physical activity among a sample of Toronto children.

**Methods:**

The sample comprised 856 grade 5 and 6 students from 18 elementary schools in Toronto, Ontario. Multilevel linear regression analyses were used to examine the impact of school physical activity policy on students’ time spent in light-to-vigorous physical activity.

**Results:**

Significant between-school random variation in objectively measured time spent in light-to-vigorous physical activity was identified [σ^2^μ_0_ = 0.067; p < 0.001]; school-level differences accounted for 6.7% of the variability in the time individual students spent in light-to-vigorous physical activity. Of the 22 school-level variables, students attending schools with support for active transportation to/from school and written policies/practices for physical activity, accumulated significantly more minutes of physical activity per school week than students who attended schools that did not.

**Conclusions:**

School physical activity policy and support for active school travel is associated with objectively measured time spent in light to vigorous physical activity. School physical activity policy might be a critical mechanism through which schools can impact the physical activity levels of their students.

## Background

Only seven per cent of Canadian children and youth are meeting the Canadian Physical Activity Guidelines for Children and Youth of at least 60 minutes of daily moderate to vigorous physical activity (MVPA) [1]. In addressing this physical inactivity pandemic, solutions will require a comprehensive, ecological approach that influences policies, physical and social environments, together with the more traditional focus of interventions targeting individual-level factors such as barriers and self-efficacy.

A key tenet of the ecological approach is that there is interaction between individuals and the settings in which they spend their time. In terms of place effects on health, Macintyre, Ellaway, and Cummins [2] make the distinction between ‘compositional’ factors (which people are found in a place) and ‘contextual’ factors (the characteristics of a place), although noting that features of both infrastructure and social functioning may influence health. The school setting is an obvious setting for intervention, given that children from all socioeconomic and ethnic backgrounds spend significant amounts of time there, and considering there are typically subsidized facilities, programs and staffing available to support physical activity promotion work. A school can play an important role in providing all students with opportunities to engage in physical activity.

A focus on the school setting is also warranted given evidence of the potential influence of the school environment on student health behaviours, [3] and some evidence that there is school-level variability in physical activity. In a recent examination of 35 schools in Denmark, intra-class correlation coefficients (ICC) for objectively assessed physical activity were reported, ranging between 0.06 and 0.18 for leisure time and school time, respectively [4]. This variability may indicate that children adjust their physical activity levels according to the broader social and environmental situations in which they find themselves [4].

One focus has been on the physical characteristics of schools. In one US study [5], the authors examined the association between school building and campus characteristics and objective measures of physical activity among 248 middle school students. Larger school campus and play areas per enrolled student were associated with increased physical activity. Variations in these characteristics were estimated as translating into walking an extra two miles weekly in some students.

There may also be broader variations in policies, programs or policies that promote physical activity. The identification of sources of this variation may in turn point the way for future intervention initiatives to promote physical activity at the school level. To do this, a ‘whole school’ approach is required that seeks to identify the influential aspects of school physical environments, policies or practices [6]. Leatherdale, Manske, Faulkner, Arbour, and Bredin [7] examined school and student level characteristics associated with self-reported physical activity among 2,379 grade 5 to 8 students attending 30 elementary schools in Ontario, Canada. There was significant between-school random variation for being moderately active (4.8% variability in the odds of being moderately active) and for being highly active (7.3% variability). Using the School Health Environment Survey (SHES) [8] to assess programs, activities, committees, facilities and guidelines surrounding physical activity in the school environment, Leatherdale and colleagues [7] found that after controlling for individual characteristics (e.g., sex, weight status, sport participation), youth were more likely to be highly active if they attended a school with established community partnerships, and more likely to be moderately active at schools where physical activity was used as reward rather than as discipline. These findings confirm that characteristics of the school environment are associated with physical activity levels of students.

The purpose of the present study was to extend the work of Leatherdale and colleagues [7] by first quantifying between-school variation in objectively measured physical activity. The second objective was to examine the relationship between school level characteristics (assessed by the SHES) and physical activity. The sample for this study comprised eighteen elementary schools located in the culturally and socially diverse city of Toronto, Canada’s largest urban region.

## Methods

### Study design

#### Participants

Data for this study was collected for Project BEAT (Built Environment and Active Transport; http://www.beat.utoronto.ca), which was a cross-sectional study examining how the built environment influences the way children travel to school in Toronto, Ontario. In January 2010, all elementary/intermediate schools in the Toronto District School Board (TDSB) with Grade 5 and 6 students (n = 469) received an invitation to participate. Out of 40 interested schools (response rate = 11.5%), sixteen schools were selected with respect to neighborhood type and socioeconomic status. Two of the schools were revisited a year later for resampling purposes. A different cohort of students participated at these two schools. Given several important school-level characteristics were found to be different at the two assessments, they were considered as new schools for the purposes of this analysis. This resulted in a total of eighteen schools. A total of 1704 students were enrolled in Grades 5 and 6 in these participating schools. Schools ranged in size from as few as 37 grade 5 and 6 students, to 160 grade 5 and 6 students, with the average size being 103 students. Data collection occurred April to June (2010), September to December (2010), and April to June (2011). To minimize seasonal effects, data collection did not take place during the winter months (mid-December to the end of March). Ethics approval was obtained from the Toronto District School Board and the University of Toronto Ethics Committee.

#### Data collection

All grade 5 and 6 students at the participating schools were invited to participate in the study. Active consent from parents was required. Participants completed a self-report survey in class and wore an accelerometer for a week. Parents of participating children also completed a survey and a travel diary. Using grade-specific enrolment information provided by the TDSB at the start of the 2009/2010 school year, the percentages of total grade 5 and 6 students who participated in this study were calculated. Participation ranged from as low as 34.4% of eligible students at one school, to as high as 95.2% at one school. The average participation rate, per school, was 61.3%. Overall, a total of 1027 from 1704 eligible parents/guardians at the 18 schools gave consent for their children to participate (boys, *n* = 478; girls, *n* = 549). Height and weight measurements were taken and accelerometer-measured physical activity data collected on a total of 1001 children. Of these children, 85.5% had at least 3 weekdays and 1 weekend day of valid data (*n* = 856; boys = 389, girls = 467). This resulted in a final response rate of 50% (i.e., 856/1704 × 100). This response rate is consistent with several previous active consent studies examining obesity and physical activity among Canadian elementary students [7,9]. At each school (n = 18), the administrator(s) most knowledgeable about the school’s programs, policies and resources were invited to complete the School Health Environment Survey (SHES).

### Measures

#### Physical activity

Children’s physical activity was objectively assessed using Actigraph Model GT1M accelerometers. Further details of the accelerometry data collection and analysis protocol are available [10]. Participants were asked to wear the accelerometers consistently, except for water-based activities, for eight consecutive days on a belt positioned over the right hip. A 5 s epoch was used to capture rapid transitions in activity typical in children [11]. For inclusion in data analysis, each child required a minimum of 10 hours of wearing time for at least three weekdays and one weekend day. Time spent at various levels of movement intensity was classified according to published thresholds in children [12] and used to determine levels of physical activity on school days.

Given the conservative cut-off points adopted in Project BEAT for moderate and vigorous activity [10], our dependent physical activity measure was average daily (week day) minutes spent in light to vigorous physical activity to increase data variability. Increasing emphasis is being placed upon exploring all accelerometer-measured intensities of physical activity in analyses, especially light activity [13] which has largely been ignored in the accelerometer literature, given health benefits accrue when sedentary time is replaced by light activity [14]. Time spent in light intensity physical activity also has favourable associations with some biomarkers (blood pressure, cholesterol) in adolescents [15]. More importantly, it is likely some environmental influences are more prone to impact light physical activity. For example, a supportive social environment may encourage incidental physical activity throughout a school day rather than facilitating structured bouts of moderate to vigorous physical activity.

### Student-level variables

Student level variables were assessed through self-report by the child [age (years); sex (female = 1, male = 0)]. Weight was measured to the nearest 0.1 kg and height was measured to the nearest 0.1 cm using a stadiometer. BMI was calculated (kg/m^2^) and age- and sex-specific BMI z-scores were calculated based on World Health Organization growth standards. Participants were also classified as non-overweight, overweight, or obese based on these same standards [16]. As a proxy for parental socioeconomic status, parents self-reported highest grade of education achieved by either parent (< university graduate = 1, university graduate = 0).

### School-level variables

Comprehensive School Health, a notion first introduced by the World Health Organization in 1995 [17] is now an internationally recognized framework for conceptualizing the health environment of schools. It is the basis of Healthy Schools policy in all provinces and territories across Canada, except Quebec, including the Foundations for a Healthy School model utilized by the Ontario Ministry of Education [18]. The SHES physical activity tool is consistent with the Ontario Ministry of Education model, measuring indicators associated with four foundational components of the school health environment: Healthy Physical Environment, Instruction and Programs, Supportive Social Environment, and Community Partnerships [7,8].

In scoring the SHES, each indicator was classified as falling within one of three phases of implementation, to correspond with the phases outlined by the Joint Consortium of School Health’s “Healthy School Continuum”. These three phases are described as such: Initiation, “falls short or exhibits extensive room for improvement in meeting the recommendations related to school capacity for physical activity”; Action, “meets the recommendations in several, but not all areas related to school capacity for physical activity, exhibits some room for improvement”; and Maintenance, “consistently meets or exceeds the recommendations related to school capacity for physical activity, encouraged to maintain the current level of commitment to supporting physical activity at school” [7]. Each of the four foundational components was also assigned an overall phase classification based on the combined responses to component indicators.

### Statistical analysis

Descriptive statistics were calculated for student-level (level 1) and school-level (level 2) variables. We used random intercept linear regression models to examine the impact of school physical policies on individual physical activity levels. The analyses were completed in four stages. First, we fitted a simple variance component model (null model) to determine whether there was significant variability in physical activity levels across elementary schools.

Second, we added the student-level variables (sex, age, weight status, wear time, and parental socioeconomic status) in the model. Age and wear time were grand mean centered to facilitate the interpretation of the results [19]. The level 1 variables were entered as fixed effects which assumes that they are related to the physical activity variable in the same way across level 2 units. The degree to which the estimated level 2 variance decreased after entering the student-level explanatory variables indicated how well the model explained the between-school variance.

Third, we examined a series of 22 nested models to determine which, if any, school physical activity policy variable was associated with physical activity levels. We tested the overall significance of each specific school physical activity policy variable in predicting physical activity using a scaled likelihood ratio test [19], comparing the log-likelihood difference in the (nested) model, which included the student-level variable, with the (comparison) model, which included the student-level variables and each specific school physical activity variable. A significant scaled likelihood ratio test would indicate that the comparison model fitted the data significantly better than the nested model. In this case, the specific school physical activity policy would be retained for inclusion in level 2 of the final multilevel model.

Finally, we added the significant school physical activity policy variables to the final multilevel model. By simultaneously examining school- and student-level variables, we were able to distinguish between associations related to the school physical activity policies versus those related to the characteristics of individual students. All statistical analyses were conducted using Mplus 7.0. Multilevel models were estimated using a maximum likelihood estimator with robust standard errors [19].

## Results

Table 1 presents the descriptive characteristics of students. Of the 856 students, 54.6% were female and 45.4% were male. Students were aged 9 to 12 years (Mean =11; SD = 0.61), 29.1% were overweight/obese, and 61.4% had a parent with a university education. Students accumulated an average of 213.2 minutes (SD = 43.1) daily of light to vigorous physical activity and the mean daily accelerometer wear time was 1014.2 minutes (SD = 187.3).

**Table 1:** Students’ sociodemographic characteristics (Toronto, Ontario; 2010-2011) (Level 1), n = 856

**Characteristic**	**Percent or mean (SD)**
**Sex**	
Female	54.6
Male	45.4
**Weight status**	
Normal weight	70.9
Overweight/obese	29.1
**Parental socioeconomic status**	
University educated	61.4
Not university educated	38.6
Age (years)	11.0 (0.6)
Physical activity (minutes/school day)	213.2 (43.1)
Accelerometer wear time (minutes)	1014.2 (187.3)

The descriptive statistics for the school-level variables are shown in Table 2. Overall, the majority of schools were in the action or maintenance phases for Community Partnerships (94.5%), Supportive Social Environment (88.9%), and Healthy Physical Environment (72.3%). By contrast, only 27.8% of schools were in the action or maintenance phases for Instruction and Programs, with the remainder in the initiation phase whereby extensive room for improvement was noted.

**Table 2:** School characteristics (Toronto, Ontario; 2010-2011) (Level 2), n = 18

**School PA policy variable**	**Initiation %**	**Action %**	**Maintenance %**
**Healthy Physical Environment**			
Student access to a variety of facilities on and off school grounds during school hours	0.0	33.3	66.7
Availability of physical activities during inclement weather	38.9	44.4	16.7
Student access to facilities and equipment outside of school hours	38.9	50.0	11.1
Support for active transportation to/from school	16.7	27.8	55.6
Overall Healthy Physical Environment score	27.8	66.7	5.6
Instruction and Programs*			
Implementation of daily PA	11.1	88.9	0.0
Time spent per week engaged in PA during physical education classes	72.2	22.2	5.6
Classes taught by a qualified physical education specialist	55.6	22.2	22.2
Availability and use of intramural/club activities	83.3	11.1	5.6
Consistency of intramural programming across grade divisions and seasons**	16.7	33.3	44.4
Overall Instruction and Programs score	72.2	27.8	0.0
**Supportive Social Environment**			
Emphasis placed on maximizing participation in PA through school programs	0.0	33.3	66.7
Incorporation of PA into other school subjects	22.2	72.2	5.6
Special recognition of students who participate in school physical activities	0.0	22.2	77.8
Formal collection of suggestions from the school community about PA at school**	44.8	11.1	38.9
Promotion of PA programs and events for students, families and school staff	5.6	44.4	50.0
Use of PA as a reward, not as discipline	22.2	50.0	27.8
Presence of written policies/practices for PA	16.7	50.0	33.3
Overall Supportive Social Environment score	11.1	77.8	11.1
**Community Partnerships**			
Support available for staff involved with PA	0.0	50.0	50.0
Connection to community resources	11.1	33.3	55.6
Overall Community Partnerships score	5.6	66.7	27.8

Notes:

**Not all schools completed all questions in this section. To maintain comparability between schools, two categories within this section have not been included in our analysis (‘Availability and use of interschool programs’ and ‘Consistency of interschool programming across seasons’).

**Totals do not add up to 100%, as one school gave insufficient data to yield a score in this category.

Table 3 shows the results for the multilevel linear regression models starting with the null or empty variance component model and adding student and school variables. Significant between-school random variation in objectively measured time spent in light-to-vigorous physical activity was identified [σ^2^μ_0_ = 0.067; p < 0.001]; school-level differences accounted for 6.7% of the variability in the time individual students spent in light-to-vigorous physical activity.

**Table 3:** Estimates for multilevel regression of week day physical activity as a function of student and school characteristics (Toronto, Ontario; 2010-2011)

	**Model 1: intercept only**			**Model 2: + student variables**			**Model 3: + school variables**		
	**Estimate**	**S.E.**	**p-value**	**Estimate**	**S.E.**	**p-value**	**Estimate**	**S.E.**	**p-value**
**Fixed effects**									
Intercept	213.70	3.02	<0.001	231.41	48.54	<0.001	214.68	53.62	<0.001
**Student-level variables**									
Sex (female = 1)				-32.45	-9.07	<0.001	-32.43	-9.02	<0.001
Age (years)				-6.88	-3.16	0.002	-7.01	-3.00	0.003
Weight status (overweight/obese = 1)				-5.10	-2.25	0.025	-5.17	-2.23	0.026
Parental SES (not university educated = 1)				2.41	0.71	0.477	1.77	0.56	0.574
Accelerometer wear time (minutes/school week)				0.07	9.63	<0.001	0.07	9.85	<0.001
**School-level variables**									
Support for active transportation to/from school									
Action^a^							17.33	5.16	<0.001
Maintenance^a^							5.11	1.41	0.159
Presence of written policies/practices for PA									
Action^a^							13.84	2.66	0.008
Maintenance^a^							6.32	1.22	0.224
**Random effects**									
School-level variance	125.95	45.52	0.006	142.63	4.18	<0.001	52.11	1.87	0.061
Student-level variance	1731.90	93.47	<0.001	1319.97	18.44	<0.001	1320.95	18.48	<0.001
Intraclass correlation	0.067								

Note. S.E = Standard error.

^a^Initiation is the reference category.

In model 2, results indicate that when student-level characteristics were added to the model, the variance at the school level increased (from 125.95 to 142.63) and remained statistically significant (p < 0.001). In this model, female, older, and overweight/obese students accumulated significantly fewer minutes of physical activity per school-week. On average, female students engaged in 32.45 fewer minutes of physical activity than did male students. Overweight/obese students had on average 5.10 fewer minutes of physical activity than students who were not overweight or obese. Age was negatively associated with physical activity; for every one unit increase in a student’s age, physical activity decreased by 6.88 minutes. Parental socioeconomic status (p = 0.447) was not significantly associated with physical activity.

Results from the scaled log-likelihood difference chi-square tests that were used to examine whether the inclusion of a specific school physical activity policy made a significant contribution to the prediction of physical activity are shown in Table 4. Of the 22 school physical activity policy variables assessed, only *Support for active transportation to/from school* (scaled *X*^2^ diff(Δ df = 2) = 9.49, p = 0.009) and the *Presence of written policies/practices for physical activity* were significantly associated with physical activity (scaled *X*^2^ diff(Δ df = 2) = 6.93, p = 0.031).

**Table 4:** **Nested model comparisons - results from the chi-square difference testing based on loglikelihood values and scaling correction factors**^**a **^**(Toronto, Ontario; 2010-2011)**

	**Loglikelihood**	**Scaling correction factor**	**Number of free parameters**	**Scaled *X***^**2 **^**difference**	**df**	**p-value**
*Nested model (model 2 of Table*3*: intercept + student-level variables)*	-4342.60	1.15	8			
*Comparison Model (model 2 of Table*3*+ each specific school PA policy indicator)*						
**Healthy Physical Environment**						
1. Student access to a variety of facilities on and off school grounds during school hours	-4305.88	1.09	9	0.18	1	0.672
2. Availability of physical activities during inclement weather	-4304.55	1.09	10	2.58	2	0.276
3. Student access to facilities and equipment outside of school hours	-4305.78	1.03	10	0.47	2	0.790
4. Support for active transportation to/from school	-4302.08	1.03	10	9.49	2	0.009
5. Overall score for this indicator	-4305.95	1.07	9	0.06	1	0.801
**Instruction and Programs**						
6. Implementation of daily PA	-4305.74	1.10	9	0.38	1	0.540
7. Time spent per week engaged in PA during physical education classes	-4305.97	1.07	9	0.02	1	0.877
8. Classes taught by a qualified physical education specialist	-4304.87	1.03	10	2.65	2	0.266
9. Availability and use of intramural/club activities	-4305.51	1.12	9	0.65	1	0.421
10. Consistency of intramural programming across grade divisions and seasons	-4303.91	1.09	10	3.71	2	0.157
11. Overall score for this indicator	-4305.94	1.09	9	0.06	1	0.810
**Supportive Social Environment**						
12. Emphasis placed on maximizing participation in PA through school programs	-4305.96	1.09	9	0.03	1	0.854
13. Incorporation of PA into other school subjects	-4305.98	1.05	9	0.01	1	0.943
14. Special recognition of students who participate in school physical activities	-4305.79	1.09	9	0.31	1	0.580
15. Formal collection of suggestions from the school community about PA at school	-4305.80	1.18	10	0.22	2	0.895
16. Promotion of PA programs and events for students, families and school staff	-4305.56	1.10	9	0.67	1	0.413
17. Use of PA as a reward, not as discipline	-4304.30	1.06	10	3.39	2	0.184
18. Presence of written policies/practices for PA	-4302.75	1.05	10	6.93	2	0.031
19. Overall score for this indicator	-4305.50	1.09	10	0.84	2	0.657
**Community Partnerships**						
20. Support available for staff involved with PA	-4303.21	1.13	9	3.66	1	0.056
21. Connection to community resources	-4305.26	1.11	10	1.14	2	0.565
22. Overall score for this indicator	-4305.41	1.04	9	1.48	1	0.224

^a^The formula for scaled chi-square difference testing for nested models was derived from: http://www.statmodel.com/chidiff.shtml (last accessed October 2, 2013).

Model 3 of Table 3 shows that when we added *Support for active transportation to/from school* and the *Presence of written policies/practices for physical activity* variables in the multilevel model, the between school variance diminished markedly (from 142.63 in model 2 to 52.11 in model 3) and was no longer statistically significant (p = 0.061). The associations that were noted between the student-level variables and physical activity in model 2 remained relatively unchanged with the addition of the school-level measures.

**Figure 1:**
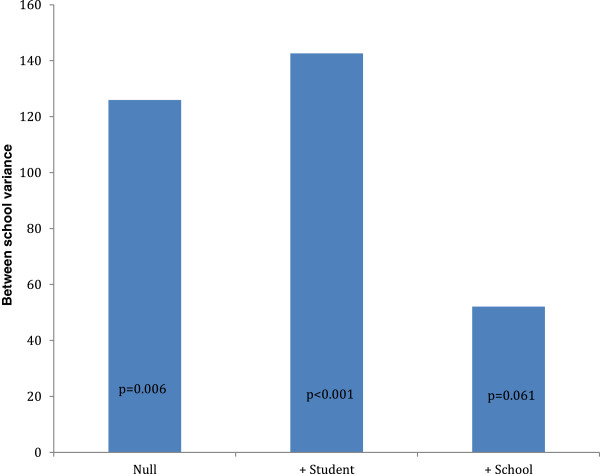
Change in between-school variance with the sequential addition of student-level and school physical activity policy variables (Toronto, Ontario; 2010-2011).

Figure 1 graphically summarizes the change in between-school variance with the sequential addition of student-level and school physical activity policy (Support for active transportation to/from school and the Presence of written policies/practices for physical activity) variables to the multilevel linear regression models for physical activity. The between school variation did not change appreciably and remained significant when the student-level variables were added. However, when *Support for active transportation to/from school* and the *Presence of written policies/practices for physical activity* variable was added, the between-school variance diminished markedly and was no longer statistically significant.

Analyses were replicated using minutes of light to vigorous physical activity during school hours (based on start and end times provided by schools – approximately 8.30 a.m. to 3.30 p.m.) and similar results were found (data available on request). The intraclass correlation was 0.103, indicating that the school-level variance contributed approximately 10% to the overall variance in physical activity. Only the *Presence of written policies/practices for physical activity* was significantly associated with physical activity (scaled *X*^2^ diff(Δ df = 2) = 8.29, p = 0.016).

## Discussion

This study confirms the potential for significant between-school variation in physical activity. Earlier research using objective measures of physical activity has reported school level ICCs ranging from 0 [20] to 0.29 [21]. In the current study, 6.7% of the overall variance in physical activity was explained by school-level variance when controlling for a limited number of individual level student characteristics. Notably, the intraclass correlation of 0.067 was similar to that reported by Kristensen and colleagues [4] in their study of 35 Danish schools using accelerometry (ICC = 0.06 for leisure time physical activity). This increased to 10% when using average daily minutes of light to vigorous physical activity on a school day as the dependent measure. The pooling of these estimates will be informative for future researchers in estimating sample sizes required for future school based physical activity interventions [4].

A critical question is: what factors are associated with this variability? Using the SHES instrument, it was demonstrated that students were more physically active at schools that supported active transportation to/from school. On average, students attending schools in the maintenance or action phases accumulated significantly more minutes of physical activity per school week: 5.11 and 17.33 minutes more, respectively, than students who attended schools in the initiation phase for this indicator. For this indicator, scores were primarily based on the various ways in which schools promote active transportation, such as through identification of safe routes in school newsletters, provision of crossing guards, designation of car-free zones around the school, policies allowing students to bring bicycles and organized walk-to-school programs and events. Reviewing SHES responses, schools in the initiation phase did not have organized walk-to-school programs and events, and the majority did not allow bikes or scooters on school property. Given the importance of the school trip as a source of physical activity [22], this finding is encouraging in highlighting the critical role schools may play in encouraging and facilitating active school travel while achieving broader public health goals related to children’s physical activity. Greater support for schools in developing and implementing strategies to promote active transportation are warranted.

Students were also more physically active at schools with established written policies/practices for physical activity both over the whole day and during the school day. For this indicator, schools were scored based on whether their priorities for physical activity in the areas of curricular education (i.e. during instructional time), intramural programs/club activities (i.e. extracurricular activities within the school) and interschool programs (i.e. competitive extracurricular activities with other schools) were outlined through existing written policies, written policies still under development, through professional practice only, or not at all. Schools with all or most of their physical activity priorities outlined through existing written policies were scored in the “maintenance” phase for this indicator. Schools with at least two of these priority areas outlined in practice only, or with some areas not outlined at all, were scored in the “action” phase, whereas those who had not outlined these priority areas at all were scored in the “initiation” phase. On average, students attending schools in the maintenance or action phases accumulated significantly more minutes of physical activity per school week: 6.32 and 13.84 minutes more, respectively, than students who attended schools in the initiation phase for this indicator.

As with the active transportation indicator, it is not clear why children were more active at schools in the action phase as opposed to the maintenance phase. Other unmeasured variables may be contributing to this difference. For example, schools in the action stage for supporting active transportation may be located in neighbourhoods that are more conducive to walking and cycling. Alternatively, the primacy of current school initiatives compared to past priorities (i.e. those in the action phase compared to those that are already in the better-established maintenance phase) may contribute to this phenomenon in that staff may be more likely to attend to issues that are more immediate.

Our findings contrasted with those of Leatherdale and colleagues [7] who found that students were more likely to be active if they attended a school that used physical activity as a reward and had established community partnerships. Differences in methodology (accelerometry in the current study versus self-reported physical activity data in the former) and sample size (18 versus 30 schools, respectively) might play a role. One commonality among these studies was the finding that the majority of schools were in the initiation phase for the overall score for Instruction and Programs (72.2% in the current study compared to 73.3% in the former). That is, a majority of schools in both studies required increasing their capacity to provide opportunities for physical activity through traditional programming such as physical education, intramural and interschool sport programming. The introduction of written policies may be one way in which schools can begin to address this gap. Developing written policies regarding physical activity demonstrates a commitment to encouraging this health behaviour, while outlining expectations of the roles and responsibilities of staff, students and parents. Such policies might also reflect on strategies that could address consistent disparities in physical activity patterns of children, particularly between boys and girls.

There is little systematic surveillance of policies addressing physical education and school sport opportunities in Ontario schools. For example, the Ontario Ministry of Education introduced Policy/Program 138: Daily Physical Activity (DPA) in October of 2005, and this was integrated into the revised Health and Physical Education curriculum document in 2010. Yet to date, there is still no comprehensive picture of policy implementation across the province. It is also interesting to note that 3 of 18 schools in the current study indicated that there are no existing written policies regarding physical activity priorities in their school, despite the fact that DPA is extant in all of these schools. Certainly, more research is required in measuring adherence to existing physical activity policies, as well as in identifying the barriers and facilitators of policy implementation for educators and school administrators.

There are a number of limitations to this study. Given the cross-sectional design, causal relationships cannot be inferred. Longitudinal research is required in examining whether changes in the school environment precede changes in physical activity levels. Schools were not sampled to ensure variability in the school setting as measured by the SHES. As data were drawn from a convenience sample of schools, we cannot infer that these results are representative of all schools in Toronto. Finally, there may be a range of other confounding effects that are not adjusted for while we also only controlled for a limited number of student level characteristics. Twenty-two school-level variables were assessed but only two were significant. Given the number of analyses, the significant findings may have occurred by chance. Future research with larger samples is needed to confirm our findings.

## Conclusion

Where a child goes to school matters for overall level of physical activity, as evidenced by significant school-level variations in objectively measured physical activity. Students were more physically active in schools that had written policies for physical activity and that supported active travel to/from school. These findings may indicate the importance of more broadly formalizing a school’s commitment to physical activity promotion through the adoption of written policies and strategies that extend beyond the school day.

## Competing interests

The authors declare that they have no competing interests.

## Authors’ contributions

The work presented here was carried out in collaboration between all authors. GF conceived of the study, conducted the analysis, and drafted the manuscript. LZ contributed to the data collection and analysis, and helped draft the manuscript. MS coordinated the data collection and contributed to analysis. SL and SM contributed to initial design and interpretation. All authors read and approved the final manuscript.
